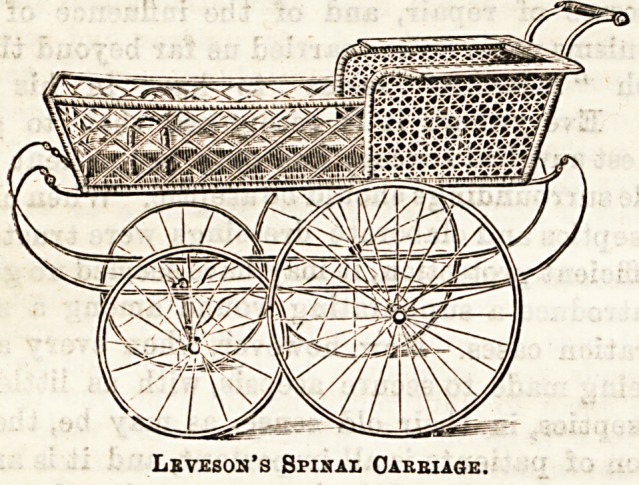# Practical Departments

**Published:** 1896-07-11

**Authors:** 


### PRACTICAL DEPARTMENTS.

### SPINAL CARRIAGE.

The spinal carriage here illustrated is designed and manufactured by Messrs. Leveson and Sons, New Oxford Street, and Knightsbridge. It is an excellent little carriage, very light and easy of movement, the body being made of fine cane-work, painted a light shade of tan colour. The cushioning is done in American leather, of the same tint, and the hood is a brass-jointed one. The "easy-riding" springs

Leveson's Spinal Carriage.

minimise jars and shakes to the occupant, and the bicycle rubber-tyred wheels complete the comfort of the carriage. The rubber tyres are known as "cementless" and cannot come off, being fixed by means of a wire in the centre of the rubber. Amongst a number of invalid carriages and chairs ordered lately from Messrs. Leveson by Lady Warwick for her "Cripples' Home" at Milverton were several of these spinal carriages. The price varies according to size, from 10 to 14 guineas. The carriage is made in all sizes, both for children and adults.

## Figures and Tables

**Figure f1:**